# 

**Published:** 1781-07

**Authors:** 


### LONDON MEDICAL JOURNAL.

BY

A SOCIETY OF PHYSICIANS.

By Dr Simmons &c.

---

VOL. II.

July – Dec 1781.

---

Surgeon Genl's Office LIBRARY. 63441 Washington, D.C.

LONDON:

Printed by WILLIAM RICHARDSON in the Strand;

AND SOLD

By J. WALKER, N° 20, Pater-noster Row.

MDCCLXXXII.

1781.

ſtitution amounts to about 100 guineas. The inſpectors are authorized to give a reward to the perſon who firſt informs them that a freſh family is infected.

---

The following new works are in the preſs; viz. 1. Obſervations on Diſorders of the Nerves. By Alexander Thomſon, M. D. 2. Elements of the Branches of Natural Philoſophy connected with Phyſic. By John Elliot, M. D.

---

### PROMOTIONS.

Dr. Matthews to be phyſician to St. George's Hoſpital in London, in the room of Dr. Giſborne, who has reſigned.—Mr. Ingle to be ſurgeon to the Infirmary at Leiceſter.—*June* 26. John Sibthorpe, M. B. of Lincoln College, Oxford, to be one of Dr. Radcliffe's travelling phyſicians.—*July* 3. Charles Davy, M. A. of Gonville and Caius College, Cambridge, to be M. B.—10. Mr. John Robertſon to be ſurgeon to Dalrymple's corps in Jamaica, in the room of Mr. Richard Allen deceaſed.—Mr. —— Wood to be ſurgeon to the 2d regiment of foot in the room of Mr. John Perryn.—Mr. James Kay to be ſurgeon to the 70th regiment regiment of foot, in the room of Mr. Lighteizer. —Mr. Phillips to be furgeon to the 85th regiment of foot, in the room of Mr. John Rutherford.—Mr. Thomas Davy of the 57th, and Mr. Donald M'Intire of the 43d regiments of foot, to be furgeons to the general hofpital in North America.

---

### DEATHS.

*June* 12. At Finchley, in Middlefex, Sir Thomas Harris, Knight, one of the court of affiftants of the Company of Apothecaries, in London.

*July* 9. At Long Melford, in Suffolk, Mr. Jofeph Howlett, formerly an apothecary in London.

20. In South Audley-ftreet, London, Mr. Wheler, apothecary.

---

### SECTION IV.

### MONTHLY CATALOGUE.

1. A Short account of the moft effectual means of preferving the Health of Seamen. 4to. New York, 1780. 20 pages.

This

This little tract, which is written by Dr. *Gilbert Blane*, physician to the fleet under Sir George Rodney, is compiled chiefly from the writings of Dr. Lind and Captain Cook. He has arranged his observations under the three general heads, of fevers, fluxes, and the scurvy. Speaking of the first of these Dr. Blane observes, that infectious fevers are not so frequent in the West Indies as in England, there being something in the tropical climate averse to the production or continuation of a disease of this kind: "I have seen," says he, "so many instances of crowding and "nastiness, in ships and hospitals, without conta-"gion being produced, and which in Europe "would have excited it or rendered it more ma-"lignant, that the fact is ascertained beyond a "doubt. Farther, those ships which bring this "infectious fever from Europe, in general get rid "of it soon after coming to this climate, and no-"thing but the highest degree of neglect can revive "it. This brought into my mind what is related "of the plague at Smyrna and other places; that "it disappeared at the hottest part of the season; "and it is a disease never heard of in the Torrid "Zone. It is very difficult to ascertain the cause "of this, as every thing relating to infection is "very obscure. We can conceive it to be owing

"to

" to the greater degree of airinefs which the heat " of the climate makes neceffary, or the employ- " ment of lefs woollen clothes; there may be " fomething in the ftate of the body, particular- " ly of its furface, which difpofes it lefs to pro- " duce or imbibe the poifonous effluvia, or more " probably the virulent matter is of fuch a de- " gree of volatility as to be readily diffipated in a " certain degree of heat."

Dr. Blane profeffes to have written his book chiefly for the ufe of officers of the navy, who though in general willing to adopt ufeful regulations for preferving the health of their fhips crew, have feldom opportunity or inclination to confult voluminous publications on phyfic. To thefe, and to the fea-faring people in general, the prefent performance will no doubt be extremely ufeful, as it comprizes, within a fmall compafs, the moft judicious remarks that have hitherto been made on the fubject.

2. An Effay on Fire. To which is added an Appendix. By *C. R. Hopfon*, M. D. octavo. *Rivington.* London, 1781. 144 pages.

The doctrine maintained by the author of this work is, that heat, as well as light, is a diftinct elementary body, having properties different from thofe of all other bodies, as well as from thofe of light; that its particles enter more or lefs less into the compofition of all bodies, and remain in a fixed or quiefcent ftate, in confequence of their attraction to the particles of fuch bodies; that when extricated or fet at liberty from thofe particles, with which they were before united, by the intervention of other bodies, to which fuch particles have a ftill ftronger attraction, as in the cafe of friction, percuffion, or commenftruation, they recover, what may be called, their original or native activity, and diffufe themfelves throughout the bodies of which they were a part: that in thefe circumftances, and in confequence of fuch activity, they have the power or property of producing the fenfation of heat, and likewife of communicating this power to thofe bodies, throughout which they are thus diffufed.——That fire therefore is not a fimple element, but is compounded of two diftinct elementary bodies, *viz.* light and heat, in a ftate of the moft intimate union or combination.

According to this opinion, phlogifton is neither pure light nor pure heat, but is compounded of both thefe fubftances, intimately combined with each other, and with the particles of thofe bodies in which it exifts: in confequence of which laft combination it lofes its activity, and becomes fixed or quiefcent in fuch bodies.

In

In the appendix the author has added his inaugural diſſertation *De tribus in uno*, which was firſt printed at Leyden in 1767.

3. Obſervations on fevers: wherein the different ſpecies, nature, and method of treating thoſe diſeaſes are repreſented in new and intereſting points of view. By *John Roberts*, ſurgeon, late of the royal navy, now of Grafton-ſtreet, Soho. 8vo. *Robinſon*, London, 1781. 2 s. 6 d.

4. An Eſſay on Culinary Poiſons. Containing cautions relative to the uſe of laurel leaves, hemlock, muſhrooms, copper veſſels, earthen jars, &c. with obſervations on the adulteration of bread and flour, and the nature and properties of water. 8vo. *Kearſley*, London, 1781. 45 pages. 1 s.

The ſuperficial account here given of the lauro-ceraſus is copied chiefly from Dr. Mead, on whoſe authority the compiler has probably been induced to ſpeak of ſal ammoniac as the antidote to this poiſon. In caſes where the diſtilled water of this plant has been ſwallowed, its fatal effects have generally come on too ſuddenly to allow of any relief from medicine; but it would ſurely be a melancholy circumſtance if any well-meaning perſon, relying on what is aſſerted in this pamphlet, ſhould, in a caſe of this kind, in-

ſtead of endeavouring to empty the ſtomach inſtantly by a powerful emetic, content himſelf with giving the patient "from ten to forty drops "of ſal ammoniac in a glaſs of water;" and yet this is the only remedy ſpoken of!

What is ſaid of ſmall hemlock or fool's parſley is comprized in about a dozen lines, which include a ſhort general deſcription of the plant. The account of muſhrooms contains a ſmall part of an ingenious paper on *fungi*, inſerted in the Gentleman's Magazine for 1755.—The cautions againſt the uſe of copper veſſels are taken from Dr. Mead's Eſſay on Poiſons, and M. Amy's Treatiſe upon ciſterns; the remarks on the ſolution of ſalt of lead from a diſſertation by Dr. Lind; as thoſe on bread and flour are from a tract on the nature of bread by Dr. James Manning, publiſhed in 1757; and the obſervations on water, which occupy the laſt ſixteen pages of the pamphlet, are copied from Dr. Rotheram's "Philoſophical Inquiry."

5. Memoire ſur les enfans trouvés; par les recteurs de l'hopital general de S. Jacques d'Aix; *i. e.* A memoir concerning foundlings, by the managers of the general hoſpital at Aix. 4to. 1780. 190 pages.

This

This paper, which appears to be written with the moſt humane views, is occaſioned by the mortality that has prevailed of late in the foundling hoſpital at Aix, in Provence. It ſeems that of one hundred and fifteen children admitted in one year, one hundred and three died. The cauſes aſſigned for this fatality are, 1. The diſeaſes that attack the children ſoon after their birth; 2. The inſalubrity of the apartments, and improper regimen; 3. The want of proper nurſes. The authors point out the means of remedying each of theſe inconveniences.

6. Monitum ad obſervatores, ſocietatis meteorologicæ Palatinæ, à ſereniſſimo electore Carolo Theodoro recens inſtitutæ. 4to. Manheim, 1781. 12 pages.

This work contains an invitation to the lovers of meteorology in different parts of Europe to cooperate with the views of the newly-eſtabliſhed ſociety, and an offer on the part of the Elector Palatine to furniſh them with the neceſſary apparatus.

7. La Chimie Domeſtique; *i. e.* Domeſtic Chemiſtry. 8vo. Lauſanne, 1780. 19 pages.

8. Eſſai ſur l'electricité, naturelle & artificielle, par M. le Comte de la Cepede, des academies de Dijon, Stockholm, Heſſe Hombourg, Mu-

 nich, nich, &c. *i. e.* An Effay on natural and artificial electricity, by Count de la Cepede, of the academies of Dijon, Stockholm, Heffe Hombourg, Munich, &c. 2 vols. 8vo. Paris, 1781.

9. Ratio occurrendi morbis à mineralium abufu produci folitis, auctore *Theodorico Petro Caels*, Collegii Medicorum Bruxellenfium focio, 12mo. Amftelodami, 1781. p. 117.

10. Analyfe des eaux minerales de S. Vincent, et de Courmayeur, dans la duché d'Aofte, avec une appendice fur les eaux de la Saxe, de Pré S. Didier, et de Fontane More; par *M. Giovanetti*, Docteur Collegié, Doyen et Vice-prieur de la Faculté de medecine de Turin, Medecin penfionnaire de S. M. Contenant plufieurs procedés chymiques nouveaux, utiles pour l'analyfe des eaux minerales en general, et pour celle des fels. *i. e.* Analyfis of the mineral waters of Saint Vincent and Courmayeur, in the duchy of Aofte, with an appendix on the waters of Saxe, Pré Saint Didier, and Fontane-More; by *M. Giovanetti*, Collegiate Doctor, Dean and Vice Prior of the Faculty of Phyfic at Turin, and Phyfician penfioned by his Majefty; containing feveral new chemical proceffes, ufeful in the analyfis of mineral waters in general, and in that of falts. 8vo. Turin, 1779.

11. Hiſtoire naturelle, chymique, et medicinale des corps des trois regnes de la nature, ou abrégé des œuvres chymiques de M. Gaſpard Neumann, par feu *M. Roux*, Docteur de la Faculté de Medecine de Paris, Profeſſeur de chemie, &c. Premiere partie du regne mineral. *i. e.* The natural, chemical, and medicinal hiſtory of the bodies belonging to the three kingdoms of nature, or an abridgment of the chemical works of Gaſpard Neumann, by the late *M. Roux*, Doctor of the Faculty of Phyſic at Paris, Profeſſor of Chemiſtry, &c. 4to. Paris, 1781. 338 pages.

This poſthumous publication forms only a ſmall part of a plan which the ingenious editor projected ſeveral years before his death. Neumann's chemical writings, with the annotations of Zimmermann and Lewis, were the groundwork upon which M. Roux intended to form a complete ſyſtem of chemiſtry. The lovers of that art will regret that he lived to finiſh only this fragment, which contains the firſt, and a part of the ſecond of the ſix claſſes into which he meant to divide the mineral kingdom. The firſt of theſe claſſes includes ſtones and earths; the ſecond, metallic ſubſtances; in the third, he propoſed to arrange ſulphur; in the fourth, mineral ſalts; in the fifth, chryſtals, and in the ſixth and laſt, the different kinds of water.

In

In his introduction to the work, M. Roux divides chemistry into natural and experimental. The former of these includes the phenomena produced by the action of bodies upon each other, by virtue of their nature and particular composition. Under this head he considers meteors, the production of fossils, earthquakes, vegetation, &c. The second branch, or experimental chemistry, includes all the phenomena which present themselves to the chemist when he himself applies these substances to each other. This part of his subject M. Roux distinguishes into, 1. Philosophical chemistry; 2. Medicinal chemistry; 3. Zimotechnicks, or the art of making bread, and the different wines; 4. Cookery; 5. The art of perfumery; 6. Pyrotechnics, or the art of making fireworks; 7. Halotechnics, or the extraction of salts, and their spirits; 8. The art of making soap; 9. That of dying; 10. That of varnishing; 11. The different arts for the preparation of skins, hair, horns, &c. 12. Those of preparing grease and tallow, including the art of making glue; 13. The art of making paper; 14. That of making starch; 15. Metallurgy, which includes the docimastic art, and that of alloys; 16. Alchymy, or the art of improving and transmuting metals; 17.

The

The art of making glafs, and that of enamelling; 18. Pottery, in which are included the arts of making tiles, bricks, varnifhed pottery, porcelaine, &c. 19. The art of preparing lime, mortar, &c.

After pointing out thefe diftinctions M. Roux treats very fully of the nature and action of fire, the conftruction of furnaces, and of the veffels and inftruments neceffary in chemiftry. He next defcribes the different modes of diftillation, together with the precautions and apparatus requifite in each. In treating this part of his fubject, he takes occafion to defcribe the different lutes that are in ufe, and to point out fuch as he thinks beft. The introduction concludes with feveral remarks relative to the care and attention to be obferved in chemical operations.

In the work itfelf M. Roux, in the firft clafs of his arrangement, defcribes with great accuracy, the experiments made of late years by M. D'Arcet and others, on the diamond and other precious ftones. He treats very minutely of quick lime, gypfum, the Bologna ftone, and fufible fpar. In defcribing the lapis lazuli, and the valuable colour with which it furnifhes painters, he mentions the proceffes employed for extracting it. To his refearches on the argillaceous laceous earths he has added ſeveral methods of compoſing excellent crucibles. He ſpeaks of the fuſion of talk in the furnaces at Saint Gobin, and of its being attended with this ſingular circumſtance, that the ſemi-vitrification produced by this fuſion did not unite with the crucible. Of the perfect metals, which conſtitute M. Roux's ſecond claſs, he completed only the articles of gold and ſilver, under each of which heads we meet with a variety of intereſting obſervations.

12. Dictionnaire raiſonné de Phyſique. Par *M. Briſſon*, de l'Academie Royale des Sciences, maitre de Phyſique et d'Hiſtoire naturelle des Enfans de France, Profeſſeur Royal de Phyſique experimentale au College royal de Navarre, et Cenſeur Royal. *i. e.* A deſcriptive Dictionary of natural Philoſophy, by *M. Briſſon*, of the Royal Academy of Sciences, teacher of natural philoſophy and natural hiſtory to the younger branches of the royal family, Profeſſor of experimental philoſophy in the Royal College of Navarre, and Cenſor Royal. 4to. Paris, 1781. Vol. I. 708 pages. Vol. II. 789 pages. With 90 copper plates.

This work includes a complete courſe of natural philoſophy, and ſeems intitled to the ſame rank in this ſcience that M. Macquer's is in chemiſtry. In a preliminary diſcourſe the author points out the order in which the principal articles ought to be read by a ſtudent.

